# Are changes in ADHD course reflected in differences in IQ and executive functioning from childhood to young adulthood?

**DOI:** 10.1017/S0033291719003015

**Published:** 2019-11-13

**Authors:** Jessica C. Agnew-Blais, Guilherme V. Polanczyk, Andrea Danese, Jasmin Wertz, Terrie E. Moffitt, Louise Arseneault

**Affiliations:** 1 Social, Genetic and Developmental Psychiatry Centre, Institute of Psychiatry, Psychology and Neuroscience, King’s College London, London, UK; 2 Department of Psychiatry, University of São Paulo Medical School, São Paulo, Brazil; 3 Department of Child and Adolescent Psychiatry, Institute of Psychiatry, Psychology and Neuroscience, King’s College London, London, UK; 4 National and Specialist Child Traumatic Stress and Anxiety Clinic, South London and Maudsley NHS Foundation Trust, London, UK; 5 Department of Psychology and Neuroscience, Duke University, Durham, NC, USA; 6 Department of Psychiatry and Behavioral Sciences, Duke University Medical Center, Durham, NC, USA

**Keywords:** ADHD, executive functioning, IQ, late-onset, longitudinal, persistence

## Abstract

**Background.:**

Attention-deficit hyperactivity disorder (ADHD) is associated with poorer cognitive functioning. We used a developmental, genetically-sensitive approach to examine intelligence quotient (IQ) from early childhood to young adulthood among those with different ADHD courses to investigate whether changes in ADHD were reflected in differences in IQ. We also examined executive functioning in childhood and young adulthood among different ADHD courses.

**Methods.:**

Study participants were part of the Environmental Risk (E-Risk) Longitudinal Twin Study, a population-based birth cohort of 2232 twins. We assessed ADHD in childhood (ages 5, 7, 10 and 12) and young adulthood (age 18). We examined ADHD course as reflected by remission, persistence and late-onset. IQ was evaluated at ages 5, 12 and 18, and executive functioning at ages 5 and 18.

**Results.:**

ADHD groups showed deficits in IQ across development compared to controls; those with persistent ADHD showed the greatest deficit, followed by remitted and late-onset. ADHD groups did not differ from controls in developmental trajectory of IQ, suggesting changes in ADHD were not reflected in IQ. All ADHD groups performed more poorly on executive functioning tasks at ages 5 and 18; persisters and remitters differed only on an inhibitory control task at age 18.

**Conclusions.:**

Differences in ADHD course – persistence, remission and late-onset – were not directly reflected in changes in IQ. Instead, having ADHD at any point across development was associated with lower average IQ and poorer executive functioning. Our finding that individuals with persistent ADHD have poorer response inhibition than those who remitted requires replication.

Children with attention-deficit hyperactivity disorder (ADHD) exhibit, on average, lower intelligence quotient (IQ) than children without ADHD (Frazier et al., 2004). Similarly, adults with ADHD tend to have lower IQ than peers without, although this difference is less pronounced than in childhood (Hervey et al., 2004; Seidman, 2006). While individuals with ADHD exhibit the full range of IQ scores and may also have high IQ, ADHD diagnosis and higher symptom levels are associated with lower IQ and poorer executive functioning on average in the population (Antshel et al., 2007). These associations are well-established cross-sectionally, however it is less clear the extent to which decreased cognitive functioning is an underlying, stable feature associated with ADHD, or a more transient manifestation of the disorder associated with concurrent expression of symptoms.

Research has found that those with ADHD in childhood tend to exhibit poorer cognitive functioning in adulthood (Seidman, 2006; van Lieshout et al., 2013; Lin and Gau, 2018), however fewer studies compare cognitive functioning between individuals whose ADHD remits *v.* those for whom ADHD persists to investigate whether remission has an ameliorative effect on cognitive functioning (Biederman et al., 2009). Neuroimaging studies suggest that remission of ADHD has a measurable impact on brain structures: while children with ADHD show cortical thinning in brain areas important for attentional processes, remission is associated with a relative thickening of these cortical areas (Shaw et al., 2013). Whether these brain changes are reflected in changes in cognitive functioning is unclear. If cognitive problems continue even when ADHD symptoms abate, this would suggest cognitive functioning is independent of overt manifestation of ADHD and unrelated to the remission process. However, if cognitive functioning improves as the disorder remits, this could indicate that aetiologic processes governing the remission of ADHD are linked with cognitive development.

Relatedly, a growing body of evidence indicates that for some individuals, ADHD may onset after childhood (Moffitt et al., 2015; Agnew-Blais et al., 2016; Caye et al., 2016; Cooper et al., 2018; Sibley et al., 2018). It is possible that late-onset ADHD is associated with emerging psychopathological processes that are reflected in worsening cognitive functioning. We took a longitudinal approach to examine whether late onset of ADHD is associated with a corresponding decline in IQ relative to unaffected peers. Examining IQ and executive functioning in this group not only helps clarify the nature of late-onset ADHD, but also provides an additional method for examining the association between cognition and ADHD symptoms, namely whether onset of symptoms is reflected in changes in IQ.

While cognitive ability, as indexed by IQ, is often thought of as stable over the life course, high correlation from age to age does not rule out individual variation over time (Ramsden et al., 2011). A correlation coefficient as high as 0.80 means nearly 40% of variation could be explained by other factors. Adolescent years are a time of particular plasticity in cognitive development: research has found a third of teenagers showed considerable variation in IQ from age 14 to 17, and this change was reflected in differences in brain volumes (Ramsden et al., 2011). The environment may play a role in IQ change: one study found a decline in IQ among children in urban Detroit, while those in suburban communities showed no decline (Breslau et al., 2001). Environmental factors such as exposure to lead or other toxins can affect cognitive ability (Reuben et al., 2017). Psychopathology is also associated with changes in cognitive ability, for example declines in cognitive functioning associated with onset of psychotic disorders and their prodromes (Reichenberg et al., 2010).

We investigated the association between ADHD, IQ and executive functioning in a population-based cohort of twins to determine whether changes in the manifestation of ADHD across development – i.e. remission, persistence and late-onset – were reflected in changes in cognitive ability. First, using a longitudinal approach, we compared trajectories of IQ among different developmental patterns of ADHD to assess whether changes in ADHD diagnosis went hand-in-hand with changes in IQ, e.g. when ADHD remits, does IQ show a relative improvement? When ADHD onsets after childhood does IQ begin to lag relative to non-ADHD peers? In this way, we can examine whether the course of IQ development differed as a reflection of change in ADHD diagnosis. Second, as the Environmental Risk (E-Risk) Longitudinal Twin Study is a cohort of twins, we were able to investigate whether the association between ADHD and IQ across development was due to a family liability shared between twins or was unique to the twin affected by ADHD, thereby shedding light on the nature of the association between ADHD and IQ. Furthermore, ADHD has been conceptualised as fundamentally a disorder of impairments in executive functioning (Brown, 2013). Therefore, third, we examined a range of tasks at ages 5 and 18 to determine whether executive functioning differed among groups with different ADHD courses from childhood to young adulthood.

## Methods

### Study cohort

Participants were members of the E-Risk Longitudinal Twin Study, a birth cohort of 2232 British children drawn from a larger birth register of twins born in England and Wales in 1994–95 (Trouton et al., 2002). Full details about the sample are reported elsewhere (Moffitt and E-Risk Study Team, 2002). The E-Risk sample was constructed in 1999–2000 when 1116 families (93% of those eligible) with the same-sex 5-year-old twins participated in home-visit assessments administered by trained research workers. All research workers had university degrees in behavioural science and experience in psychology, anthropology or nursing. Each research worker completed a formal 15-day training programme on either the mother interview protocol or the child assessment protocol to attain certification to a rigorous reliability standard. The study sample comprised 56% monozygotic (MZ) and 44% dizygotic twin pairs; sex was evenly distributed within zygosity (49% male). Families were recruited to represent the UK population with newborns in the 1990s on the basis of residential location throughout England and Wales and mother’s age; teenaged mothers with twins were over-selected to replace high-risk families who were selectively lost to the register through non-response. Older mothers having twins via assisted reproduction were under-selected to avoid an excess of well-educated older mothers. At follow-up, the study sample represented the full range and prevalence rates of socioeconomic levels in the UK (Odgers et al., 2012). Childhood SES reflected a composite of parental income, education and occupation.

Follow-up home visits were conducted when children were aged 7 (98% participation), 10 (96%), 12 (96%), and 18 years (93%). Home visits at ages 5–12 years included assessments with participants and their mother; we conducted full interviews with participants only at age 18 (*n* = 2066). There were no differences between those who did and did not take part at age 18 on socioeconomic status when the cohort was initially defined (χ^2^ = 0.86, *p* = 0.65), age-5 IQ (*t* = 0.98, *p* = 0.33), or rates of childhood ADHD (χ^2^ = 2.12, *p* = 0.71). The Joint South London and Maudsley and the Institute of Psychiatry Research Ethics Committee approved each phase of the study. Parents gave written informed consent and twins gave assent between 5 and 12 years and then written informed consent at age 18.

### Childhood ADHD diagnoses

Mothers were interviewed by research workers and asked explicitly about each ADHD symptom during home visits. Children’s symptoms were assessed with 18 items covering inattention, impulsivity and hyperactivity derived from the Diagnostic and Statistical Manual of Mental Disorders (DSM)-IV symptoms of ADHD and the Rutter Child Scales (e.g. ‘inattentive, easily distracted’, ‘impulsive, acts without thinking’, ‘very restless, has difficulty staying seated for long’). Symptoms were reported for the preceding 6 months and each symptom was scored as ‘not true’, ‘somewhat or sometimes true’ and ‘very true or often true’. Symptoms were counted as present if scored ‘very true or often true’. Teachers responded to mailed questionnaires and rated participants using the same items. Internal consistency reliabilities of parent and teacher reports were 0.90 and 0.93, respectively. Childhood ADHD diagnoses were based on DSM-IV criteria: participants had to have six or more symptoms of inattention or six or more symptoms of hyperactivity-impulsivity reported by mothers or teachers in the past 6 months, and the other informant must have endorsed at least two symptoms (thereby meeting the diagnostic criteria for the presence of symptoms in more than one setting). We considered participants to have a diagnosis of childhood ADHD if they met criteria at age 5, 7, 10 or 12. At age 5, 6.8% of participants (*n* = 131) met criteria for ADHD, 5.4% at age 7 (*n* = 102), 3.4% at age 10 (*n* = 65) and 3.4% at age 12 (*n* = 64).

### Age-18 ADHD diagnosis

We ascertained ADHD diagnosis at age 18 based on private structured interviews administered by trained interviewers with participants regarding 18 symptoms of inattention and hyperactivity–impulsivity reflecting DSM-5 ADHD symptoms (Agnew-Blais et al., 2016). ADHD diagnoses were made based on DSM-5 criteria: participants had to endorse five or more inattentive and/or five or more hyperactivity–impulsivity symptoms to be diagnosed. We also required that symptoms interfere with individual’s life at ‘home, or with family and friends’ and at ‘school or work’, thereby meeting impairment and pervasiveness criteria. The requirement of symptom onset prior to age 12 was met if parents or teachers reported more than two ADHD symptoms at any childhood assessment. A total of 8.1% of participants (*n* = 166) met criteria for ADHD at age 18.

### Remitted, persistent and late-onset ADHD groups

We identified three groups of individuals with ADHD across childhood and young adulthood: 9.5% of participants (*n* = 193) showed remitted ADHD (met diagnostic criteria in childhood but not at age 18), 2.6% (*n* = 54) persistent ADHD (met diagnostic criteria in childhood and age 18) and 5.5% (*n* = 112) ‘late-onset’ ADHD (did not meet diagnostic criteria in childhood but did at age 18) (Agnew-Blais et al., 2016). A total of 82.4% of participants (*n* = 1681) did not meet criteria for ADHD in childhood or adulthood.

### IQ and executive functioning assessment

IQ was assessed at ages 5, 12 and 18. At age 5, assessment was based on a short form of the Wechsler Preschool and Primary Scale of Intelligence-Revised (WPPSI-R) (Wechsler, 1990), prorated with the Sattler approach using Vocabulary and Block Design subtests (Sattler, 1992). At age 12, IQ was assessed with the Wechsler Intelligence Scale for Children-Revised (WISC-R) (Wechsler, 2003) and at age 18 the Wechsler Adult Intelligence Scale-IV (WAIS-IV) (Wechsler, 2008); total IQ was estimated using the same subtests – Information and Matrix Reasoning – which were pro-rated to obtain total IQ. IQ across ages was moderately correlated: age-5 IQ with age-12 IQ at *r* = 0.55 and with age-18 at *r* = 0.49, and age-12 IQ with age-18 IQ at *r* = 0.70. ADHD symptoms were negatively correlated with IQ at each age: *r* = −0.21 at age 5, *r* = −0.24 at age 12, and *r* = −0.15 at age 18 (all *p* < 0.001).

Executive functioning was assessed at age 5 and was measured as a composite mean score of the Mazes Task (Wechsler, 1990), Day–Night Task (Gerstadt et al., 1994) and Sentence Working Memory Task (Case et al., 1982). At age 18, it was assessed with the Cambridge Neuropsychological Test Automated Battery (CANTAB) (Sahakian and Owen, 1992), a computerised test battery of neuropsychological functioning that study participants completed on an iPad. Tests included Rapid Visual Information Processing (RVP); A-prime taps into sustained attention, and false alarm assesses response inhibition (due to the distribution of the data, this was dichotomised as more than two false alarms *v.* zero or one). Tests also included spatial working memory (SWM), which assesses problem-solving and the ability to hold information about spatial location in active memory, and Spatial Span (SSP), a non-verbal equivalent of the Digit Span working memory task.

### Statistical analyses

We compared IQ at ages 5, 12 and 18 among those with remitted, persistent and late-onset ADHD to those who never had ADHD. We used logistic regression to compare groups adjusting for sex and childhood SES. To understand whether changes in ADHD diagnosis across development – remission, persistence and late-onset – were associated with changes in IQ over time, we modelled IQ trajectories with multilevel regression analyses using the ‘mixed’ command in STATA (STATA, 2015). All models included random intercepts and slopes, and fixed effects for sex, childhood SES, and ADHD group. A significant ADHD group effect indicates a difference in *level* of IQ across development; a significant ADHD group by age interaction indicates a different *slope*, or trajectory, of IQ development between ADHD group and controls. We restricted analyses to individuals with information on childhood and young adult ADHD, and IQ for at least two of the three assessments (*N* = 2038).

To determine whether lower IQ among those with ADHD was accounted for by family-level factors or was unique to the twin affected by ADHD, we modelled IQ over time using multilevel regression analyses and estimated a family-wide effect, representing variation accounted for by factors common to both twins in a concordant pair, and a unique effect, representing variation specific to the ADHD twin in a discordant pair. We conducted analyses first with all twins, then with MZ twins only, to test whether any unique effect remained over and above genetic relatedness between MZ twins. We tested for consistency of these effects across time by including an interaction term between age and between- and within-twin effects.

Regarding executive functioning, we compared those with remitted, persistent and late-onset ADHD to those who never had ADHD on executive functioning composite score at age 5 and on CANTAB tasks of working memory and response inhibition at age 18 using logistic regressions adjusting for sex and childhood SES. We calculated effect sizes comparing each ADHD group to non-ADHD controls and ADHD groups to one another, adjusting only for sex and SES for the composite score at age 5, and then further adjusting for age-5 executive functioning and age-18 IQ for CANTAB task scores. All analyses adjusted for the non-independence of twin observations using the Sandwich variance estimator in Stata (STATA, 2015).

In sensitivity analyses we further explored whether those with ADHD limited to early childhood (at ages 5 and/or 7 only, *N* = 96) may have a different trajectory of IQ, as earlier recovery could be reflected in an accelerated rate of catch-up in IQ in this early childhood limited group.

## Results

### Differences in IQ amongst groups with different ADHD courses

Findings indicated that IQ does not mirror changes in ADHD course across development. Mean total IQ at ages 5, 12 and 18 was lowest in those with persistent ADHD followed by the remitted and late-onset groups, compared with controls (Table 1). IQ in the remitted group remained nearly half a standard deviation lower than controls by age 18. However, those who remitted had higher IQ scores than those who persisted: the remitted group had higher total and performance IQ at age 5, and higher verbal IQ at ages 12 and 18 compared to the persistent group. At each assessment, effect sizes comparing the remitted group to controls were smaller than those for the persistent group. The late-onset ADHD group’s total, performance and verbal IQ were lower than controls at ages 5, 12 and 18, but were also higher than those with persistent ADHD.

### Longitudinal trajectories of IQ amongst ADHD groups

**Fig. 1.**
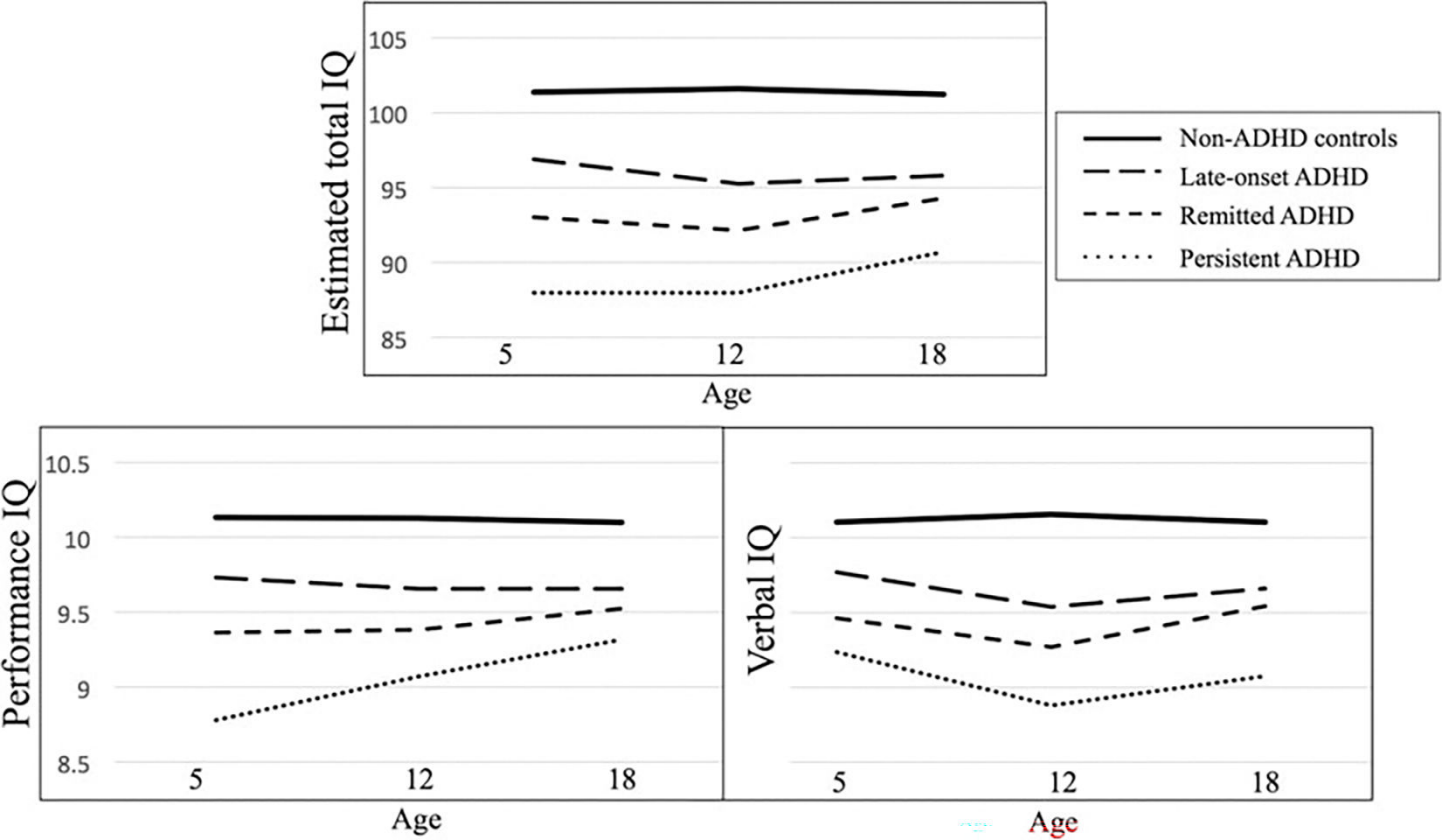
Mean total, performance and verbal IQ at ages 5, 12 and 18 amongst ADHD groups and controls.

Examining trajectories of IQ supports the finding that levels of cognitive ability in ADHD are stable from early childhood to young adulthood, even among those for whom the disorder remitted or emerged after childhood (Fig. 1). Persistent, remitted and late-onset ADHD groups all showed lower levels of IQ compared to controls, as evidenced by a significant effect of ADHD group in longitudinal models (Table 2). However, we did not find significant group by age interactions, indicating no differences in total IQ trajectory between ADHD groups and controls. Furthermore, deficits in IQ among ADHD groups were of similar magnitudes across childhood into young adulthood. Compared with persisters, the remitted ADHD group had higher total IQ across development, but their developmental trajectories did not differ. The late-onset group had intermediate IQ level between the other ADHD groups and controls, and again there was no difference in developmental trajectory of IQ. Overall, this pattern was similar for performance and verbal IQ. However, we observed an age by group interaction for performance IQ comparing persistent ADHD to non-ADHD controls, indicating the persistent group had a faster rate of development from age 5 to age 12 compared to controls. This finding suggests the persistent ADHD group may be catching up more rapidly from their larger deficit in early childhood.

### Association between IQ and ADHD controlling for familial factors

Parsing whether lower IQ among those with ADHD was entirely accounted for by family-level (between-twin pair) factors, *v.* factors unique to twins affected by ADHD (within-twin pair), led to two notable findings. First, ADHD was associated with about 10 points lower IQ across childhood and into young adulthood owing to family-wide factors (Table 3). Within-twin pair factors also accounted for lower IQ, although of smaller magnitude. In other words, when comparing families with twins concordant for ADHD with families in which both twins didn’t have ADHD, the pair with ADHD had, on average, 10 points lower IQ than the pair without; when comparing one twin with ADHD to their co-twin without, the twin with ADHD had about 1.6 points lower IQ across development. Second, when restricting analyses to MZ twins, the difference in IQ across development comparing the twin with ADHD to their co-twin without remained statistically significant, indicating the association was not entirely due to genetic factors. We found a significant interaction between the within-twin factor and age, indicating that among discordant MZ twins, IQ differed more as twins aged.

### Executive functioning amongst ADHD groups

**Fig. 2.**
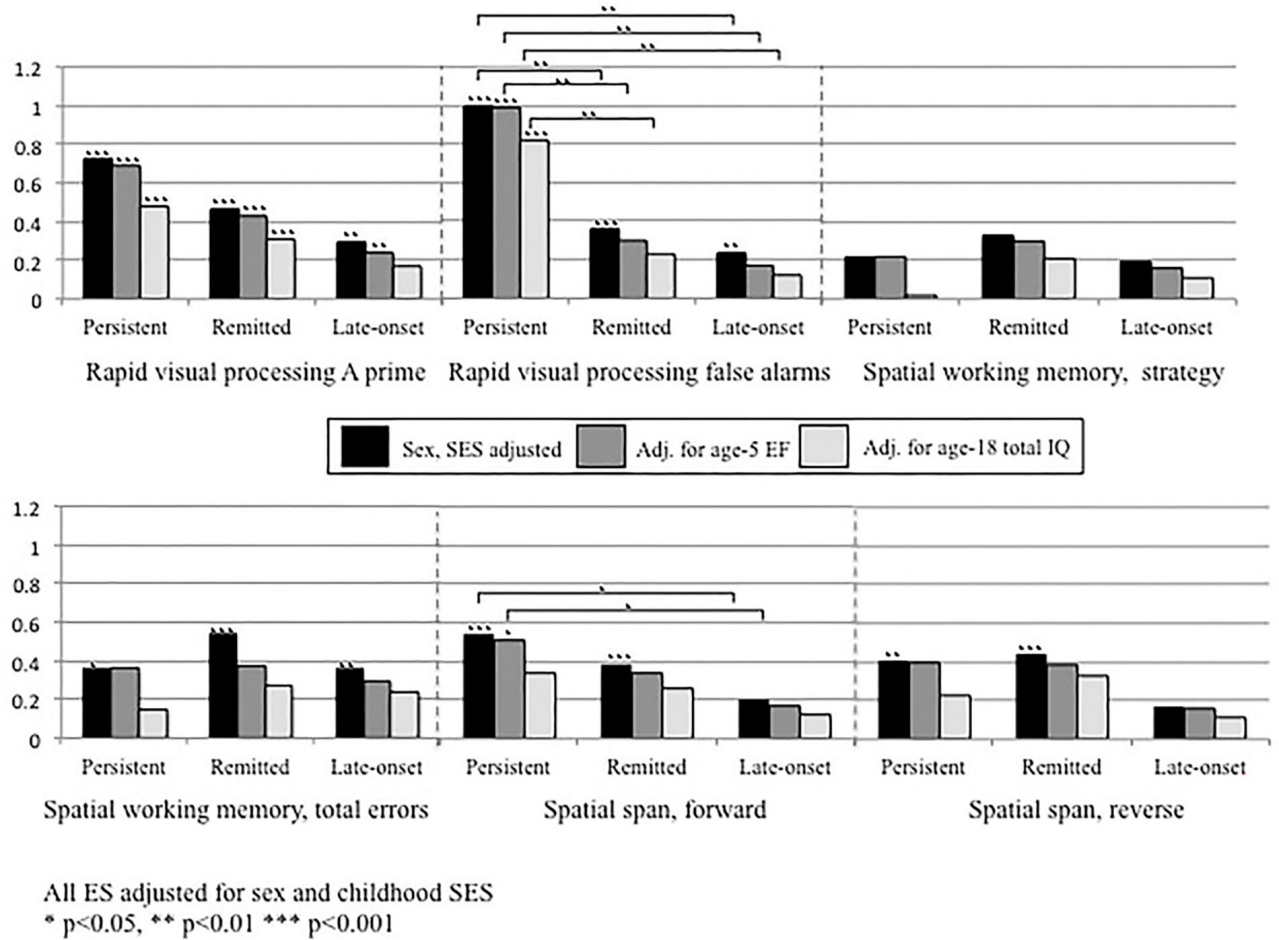
Effect sizes comparing CANTAB executive functioning task scores among those with persistent, remitted and late-onset ADHD to controls without ADHD, first adjusted for sex and childhood social class only, followed by further adjustment for age-5 executive functioning composite score, and then by age-18 total IQ. All ES adjusted for sex and childhood SES. *p < 0.05, **p < 0.01, ***p < 0.001.

At age 5, all ADHD groups had lower composite executive functioning scores than controls. However, this measure did not significantly distinguish amongst the different ADHD groups (Table 4). Executive functioning at age 18 did not appear to be sensitive to changes in ADHD diagnosis into young adulthood, as all ADHD groups performed more poorly compared with controls. Again, overall these tasks did not distinguish between the different ADHD groups. One exception was the remitted group had fewer RVP false alarms than the persistent group. Effect sizes comparing persistent and remitted groups to controls were generally of similar magnitude, while the late-onset group showed smaller effect sizes (Fig. 2). Further adjusting these comparisons for age-5 executive functioning only slightly reduced the magnitude of effect sizes, while adjusting for age-18 IQ more substantially reduced effect sizes. However, the finding that those with persistent ADHD made more RVP false alarms than those with remitted ADHD was robust to these adjustments and remained significant.

### Sensitivity analyses

Similar to the main analyses, changes in ADHD diagnosis were not reflected in changes in IQ amongst the group with ADHD limited to early childhood (online Supplementary table and figure). Similar to other ADHD groups, this early-childhood limited ADHD group exhibited a stable deficit in IQ across development.

## Discussion

We did not find that changes in the outward manifestation of ADHD – namely the disorder’s remission, persistence and late-onset from early childhood to young adulthood – were reflected in changes in IQ and executive functioning. Rather, the associations between ADHD and IQ were stable across development and independent of whether ADHD was evident at a particular developmental stage. Moreover, the association between ADHD and IQ across development was not entirely accounted for by genetic factors. Executive functioning in young adulthood did not appear to be particularly sensitive to changes in ADHD, with the possible exception of inhibitory control.

### Changes in ADHD across development not mirrored in IQ changes

While neuroimaging research suggests remission of ADHD has an impact on brain structures (Shaw et al., 2013), we did not observe that remission of ADHD by young adulthood was reflected by changes in IQ. Despite improvements in the manifestation of ADHD among those who remitted, this group exhibited a stable deficit in IQ compared with controls. While those with remitted ADHD fared better than those who persisted, longitudinal analyses showed that higher IQ among children whose ADHD remitted by age 18 was already apparent in early childhood. Among studies that have examined IQ among persisters and remitters in adolescence/young adulthood, several found no differences in IQ (Barkley and Fischer, 2011; Mick et al., 2011). One study in a clinical cohort found those with persistent ADHD had lower IQ in young adulthood compared to those who remitted, but this was also the case at childhood baseline (Cheung et al., 2016). Longitudinal trajectory analyses from a clinical cohort found remitted and persistent groups had lower IQ from middle childhood to young adulthood, but found no group difference in IQ between persisters and remitters, and no group by age interaction between ADHD groups and controls (Biederman et al., 2009). This stable deficit in IQ among those with remitted ADHD could contribute to the poorer functional outcomes experienced by individuals in this group as they reach adulthood (Agnew-Blais et al., 2018).

### Late-onset ADHD associated with lower IQ across development

Late-onset ADHD remains an area of active research, with controversy around levels of childhood psychopathology experienced by those with late-onset ADHD, and the possibility that some ADHD symptoms in this group could be explained by other disorders such as depression or anxiety (Asherson and Agnew-Blais, 2019). Given the lack of research and remaining open questions about late-onset ADHD, it is important to examine cognitive functioning in this group to better understand the nature of late-onset ADHD. If late-onset ADHD represents an emerging psychopathology, perhaps this comes along with an increasing lag in IQ behind peers as ADHD emerges. Our findings, however, do not support this hypothesis. Despite the late-onset group exhibiting lower IQ at age 18 compared with controls, it was not that the emergence of ADHD was associated with deleterious effects on IQ, but rather those who developed late-onset ADHD had lower IQ already at age 5. While slightly lower IQ may be a vulnerability factor for late-onset ADHD, this group had significantly higher IQ than those with childhood ADHD. These findings are similar to those found in the ALSPAC study, which reported that IQ of the late-onset group fell slightly below those with low ADHD symptoms across development, but also above those with persistent ADHD at ages 8 and 16 (Cooper et al., 2018). While much remains to be understood about late-onset ADHD, in the current study we find that those who meet ADHD criteria at age 18 but not in childhood show slight IQ and executive functioning deficits compared to non-ADHD controls; these deficits are present already in early childhood.

### Associations between ADHD and IQ across development not entirely accounted for by shared genetics

Our findings that ADHD and IQ share significant genetic aetiology is consistent with molecular genetic research showing higher ADHD genetic risk is associated with lower IQ and poorer working memory in childhood (Martin et al., 2015). However, while we found genetic risk explained a large portion of the association between IQ and ADHD across development (i.e. within twin pair differences were much smaller than between-pair), it did not entirely explain the longitudinal association of ADHD with lower IQ. We found differences in IQ between discordant MZ twins actually increased as the twins grew into young adulthood, which warrants further investigation. While MZ twins share nearly 100% of their DNA, they can differ, for example, in number of copy number variants and epigenetic profiles (Chen et al., 2018). These differences could lead to ADHD in one twin only, as well as to a different trajectory of IQ in the affected twin compared to their unaffected co-twin.

### Executive functioning not sensitive to changes in ADHD course

Consistent with a growing body of research (Biederman et al., 2009), we did not find that those with persistent ADHD had poorer executive functioning than those with remitted ADHD. While executive dysfunction is considered a core feature of ADHD, similar to IQ it did not vary with changes in ADHD diagnosis. Rather, having ADHD at any point in development was associated with poorer executive functioning both at ages 5 and 18. We did not find evidence for differences in working memory tasks between persisters and remitters, in contrast to some (Halperin et al., 2008; Coghill et al., 2014), but not all (Biederman et al., 2009; Cheung et al., 2016) previous studies.

We did, however, find evidence for better inhibitory control among those with remitted ADHD, as shown by fewer false alarms on the RVP task, suggesting those whose ADHD persisted may be more likely to ‘act without thinking’. This difference was robust to adjustment for age-5 executive functioning and age-18 IQ. Studies have found that individuals with ADHD perform more poorly on tests of inhibitory control, for example in Stop-Signal and Go/No-Go paradigms, and neuroimaging studies identified differential patterns of functioning in the frontal lobes during inhibitory tasks among ADHD participants and controls (Mulligan et al., 2011). Our findings suggest that inhibitory control could be of particular salience to ADHD remission. Similar results have been found by some clinical cohorts in which persistence was associated with more false alarms and fewer correct hits (Halperin et al., 2008) and more commissions on inhibitory control tasks (Fischer et al., 2005), or in which reductions in symptoms of hyperactivity-impulsivity were associated with decreased Continuous Performance Task commissions over time (Miller et al., 2013). This is consistent with functional magnetic resonance imaging findings in which the persistent ADHD group showed reduced inferior frontal cortex activity specifically during failed inhibition *v.* remitted and never ADHD groups, who did not differ from one another (Szekely et al., 2017). It is therefore possible that inhibitory control taps into a process associated with remission of ADHD. However, not all studies have replicated this finding (Vaughn et al., 2011), or have found that overall IQ explained differences in response inhibition (Lin et al., 2014). Additionally, whether those who would remit already had better inhibitory control in early childhood remains unclear.

### Limitations

This study has several strengths, including repeated assessments of IQ and ADHD spanning early childhood to young adulthood in a population-based cohort of twins. Nevertheless, some limitations must be considered. First, age-18 ADHD diagnoses were based on self-report. However, prior work in this cohort found co-informant reports of ADHD symptoms at age 18 corroborated self-reports, as those with self-reported late-onset ADHD have significantly more co-informant-rated ADHD symptoms than those without ADHD (Agnew-Blais et al., 2016). Second, our sample comprised twins and results may not generalise to singletons. Nevertheless, the prevalence of ADHD at each age in our cohort is well within ranges estimated in other samples. Third, because we assessed executive functioning at only at ages 5 and 18, we could not investigate longitudinal trajectories among the different ADHD groups. Fourth, we did not assess participants for performance on other measures associated with ADHD, such as other measures of executive functioning (e.g. Stockings of Cambridge) (Lin and Gau, 2017), response time variability, delay aversion or biometric assessments such as electroencephalogram (Michelini et al., 2016), which could be more sensitive to changes in ADHD. Fifth, we examined the association of changes in ADHD diagnosis from childhood to young adulthood (ADHD remission, persistence and late-onset) with IQ and executive functioning, rather than focusing on changes in number of ADHD symptoms over time. By concentrating on changes in ADHD diagnosis our analyses emphasise clinically important change in level of ADHD symptoms, as well as aspects of ADHD including pervasiveness of symptoms and impairment. Finally, our findings do not reveal the directionality of the underlying causal link between ADHD and IQ: it could be improvements in IQ and executive functioning affect ADHD, ADHD affects IQ, or underlying brain developments account for changes in both.

Our study emphasises the importance of taking a developmental approach to investigating ADHD and associated IQ. Lack of change in IQ in groups whose ADHD changed across development suggests that rather than lower IQ being an epiphenomenon that emerges as a by-product of ADHD symptoms, lower IQ is a stable trait associated with the presence of ADHD at any point in development. That early childhood IQ was associated with a poorer course of ADHD – both for persistence among children with ADHD and for risk of late-onset ADHD among children without – could have implications for directing additional resources to children more likely to have a more pernicious course of the disorder. Continued cognitive problems among those whose ADHD remitted may lead to poorer functioning in young adulthood, suggesting remission from the full disorder should not be considered the end to potential intervention and clinical attention. Research should continue to explore the genetic underpinnings of the association between ADHD and IQ across development, as well as potential environmental factors that might contribute to both ADHD and lower IQ. It is important that future studies follow participants past young adulthood into mid- and later-life to better understand the longer-term associations between ADHD and cognitive functioning.

## Supplementary Material

supplemental materials

**Supplementary material.** The supplementary material for this article can be found at https://doi.org/10.1017/S0033291719003015.

## Figures and Tables

**Table 1.** Total, performance and verbal IQ in childhood, adolescence and young adulthood among individuals who never had ADHD, and persistent, remitted and late-onset ADHD groups

	Never ADHD	Persistent ADHD		Remitted ADHD		Late-onset ADHD		Persistent v. remitted	Persistent v. late-onset
	*N* = 1679	*N* = 54		*N* = 193		*N* = 112			
	Mean (s.d.)	Mean (s.d.)	*d*	Mean (s.d.)	*d*	Mean (s.d.)	*d*	*d*	*d*
Childhood									
Age-5 total IQ	101.38 (14.6)	87.96 (14.7)	0.80***	93.04 (14.6)	0.44***	96.91 (15.7)	0.20**	0.34*	0.62**
Performance	10.13 (1.5)	8.78 (1.6)	0.84***	9.36 (1.4)	0.43***	9.73 (1.5)	0.19**	0.40*	0.64**
Verbal	10.10 (1.5)	9.24 (1.4)	0.49***	9.46 (1.5)	0.32***	9.77 (1.6)	0.14*	0.15	0.38*
Adolescence									
Age-12 total IQ	101.62 (14.1)	87.99 (18.3)	0.84***	92.15 (16.4)	0.54***	95.26 (15.7)	0.32**	0.29	0.55*
Performance	10.13 (1.4)	9.07 (2.1)	0.64**	9.38 (1.7)	0.41***	9.66 (1.6)	0.25*	0.21	0.41
Verbal	10.15 (1.5)	8.88 (1.4)	0.80***	9.27 (1.5)	0.52***	9.54 (1.5)	0.31***	0.27*	0.52**
Young adulthood									
Age-18 total IQ	101.21 (14.6)	90.70 (15.7)	0.64***	94.31 (15.8)	0.40***	95.80 (15.2)	0.26**	0.24^∼^	0.41**
Performance	10.10 (1.4)	9.32 (1.5)	0.46***	9.52 (1.8)	0.31**	9.66 (1.6)	0.24*	0.15	0.25
Verbal	10.10 (1.5)	9.07 (1.5)	0.66***	9.54 (1.4)	0.34***	9.66 (1.5)	0.19*	0.31*	0.52**

IQ, intelligence quotient; *d*, Cohen’s *d*; s.d., standard deviation.

Age-5 total IQ was estimated using the Sattler approach with WPPSI-R Vocabulary and Block Design subtests; age-12 and age-18 total IQ was estimated using the Sattler approach with the WISC-R (age-12) and WAIS-IV (age-18) Information and Matrix Reasoning subtests.

Total IQ scores were standardised on the full sample to mean = 100, standard deviation (s.d.) = 15, and performance and verbal IQ scores to mean = 10, s.d. = 3.

ES and *p* values adjusted for sex and childhood SES.

∼ *p* < 0.10

* *p* < 0.05

** *p* < 0.01

*** *p* < 0.001.

**Table 2.** ADHD group and group-by-age interaction effects on total, performance and verbal IQ at ages 5, 12 and 18 years comparing persistent, remitted and late-onset ADHD groups to those without ADHD

	Persistent ADHD				Remitted ADHD		Late-onset ADHD			Persistent v. remitted			Persistent v. late-onset		
	*β*	s.e.	*p*	*β*	s.e.	*p*	*β*	s.e.	*p*	*β*	s.e.	*p*	*β*	s.e.	*p*
Total IQ															
Group effect	−11.93	2.02	**<0.001**	−6.85	1.06	**<0.001**	−3.88	1.15	**<0.001**	−4.92	2.12	**0.02**	−8.58	2.46	**<0.001**
Group × age effect	0.13	0.16	0.41	0.08	0.09	0.40	0.11	−0.11	0.33	0.03	0.17	0.88	0.22	0.19	0.24
Performance IQ															
Group effect	−1.02	0.21	**<0.001**	−0.58	0.11	**<0.001**	−0.34	0.12	**0.004**	−0.47	0.22	**0.037**	−0.73	0.25	**0.004**
Group × age effect	0.04	0.02	**0.041**	0.01	0.01	0.21	−0.01	0.01	0.67	0.02	0.02	0.30	0.04	0.02	0.059
Verbal IQ															
Group effect	−0.96	0.17	**<0.001**	−0.58	0.09	**<0.001**	−0.31	0.10	**0.003**	−0.35	0.18	**0.047**	−0.71	0.21	**0.001**
Group × age effect	−0.02	0.02	0.279	0.00	0.01	0.74	−0.01	0.01	0.33	−0.02	0.02	0.24	−0.01	0.02	0.80

Bold values are statistically significant.

**Table 3.** Family-wide and unique effects of ADHD on IQ from age 5 to 18

	All twins *N* = 2015	MZ twins *N* = 1144
Family-wide effect	−10.58*** (−13.15 to −8.02)	−10.31*** (−13.62 to −7.00)
Unique effect	−1.62*** (−2.34 to −0.91)	0.62 (−1.94 to −0.03)
Unique effect interaction × Age	ns	−0.16* (−0.29 to −0.04)

CI, confidence interval; MZ, monozygotic.

Family-wide indicates between-twin pair difference; unique, within-twin pair difference. Total number of discordant twin pairs=211; number of MZ discordant twin pairs = 91.

∼ *p* < 0.10

* *p* < 0.05

** *p* < 0.01

*** *p* < 0.001.

**Table 4.** Executive functioning at age 5 and age 18 among never ADHD and persistent, remitted and late-onset ADHD groups

	Never ADHD *N* = 1679	Persistent ADHD *N* = 54	Remitted ADHD *N* = 193	Late-onset ADHD *N* = 112	Persistent *v*. remitted ES	Persistent *v*. late-onset ES
Age 5						
Executive functioning composite score	11.84 (3.0)	10.64 (3.4)*	10.54 (3.3)***	10.97 (2.8)**	0.02	0.06
Age 18 CANTAB tasks	Mean (s.d.)	Mean (s.d.)	Mean (s.d.)	Mean (s.d.)	*d*	*d*
RVP A-prime	0.89 (0.05)	0.85 (0.06)***	0.86 (0.06)***	0.87 (0.06)**	0.25	0.39*
RVP total false alarms 2+, % (N)	9.24 (15)	37.25 (19)***	5.18 (29)**	14.3 (16)	0.69**	0.71**
SWM strategy	30.60 (6.1)	31.75 (5.8)	32.43 (6.3)***	31.97 (6.4)^~^	0.12	0.04
SWM total errors	20.75 (16.3)	27.0 (19.8)*	27.85 (19.4)***	27.5 (20.8)**	0.05	0.05
SSP length, forward	6.75 (1.4)	6.02 (1.5)***	6.24 (1.5)***	6.41 (1.5)^~^	0.15	0.38*
SSP length, reverse	5.89 (1.5)	5.35 (1.3)**	5.32 (1.4)***	5.58 (1.4)^~^	0.04	-0.23

ES, effect size; s.d., standard deviation; *d*, Cohen’s *d*; RVP, rapid visual processing; SWM, spatial working memory; SSP, spatial span.

Age-5 executive functioning composite score based on mean score of the Mazes Task (Wechsler, 1990), Day-Night Task (Gerstadt et al., 1994) and Sentence Working Memory Task (Case et al., 1982).

ES and *p* values adjusted for sex and childhood SES.

~ *p* < 0.10

* *p* < 0.05

** *p* < 0.01

*** *p* < 0.001.
