# Supplementary material for: Are changes in ADHD course reflected in differences in IQ and executive functioning from childhood to young adulthood?
Source: Psychol Med. Author manuscript; Available in PMC 2026 Mar 12. (PMC12981699; doi:10.1017/S0033291719003015)

Supplemental Table and Figure. Sensitivity analyses including persistent ADHD, early childhood limited ADHD (ADHD at ages 5 and 7 only, n=96) and non-ADHD controls

|  | Persistent  ADHD | | | | Early childhood limited ADHD | | | Persistent  vs early childhood limited | | |
| --- | --- | --- | --- | --- | --- | --- | --- | --- | --- | --- |
| Total IQ | Beta | SE | p | Beta | | SE | p | Beta | SE | p |
| Group effect | -13.98 | 4.8 | **0.003** | -4.36 | | 1.48 | **0.003** | -10.4 | -2.5 | **0.012** |
| Group x age effect | 0.30 | 0.27 | 0.265 | 0.19 | | 0.14 | 0.168 | 0.05 | 0.30 | 0.874 |


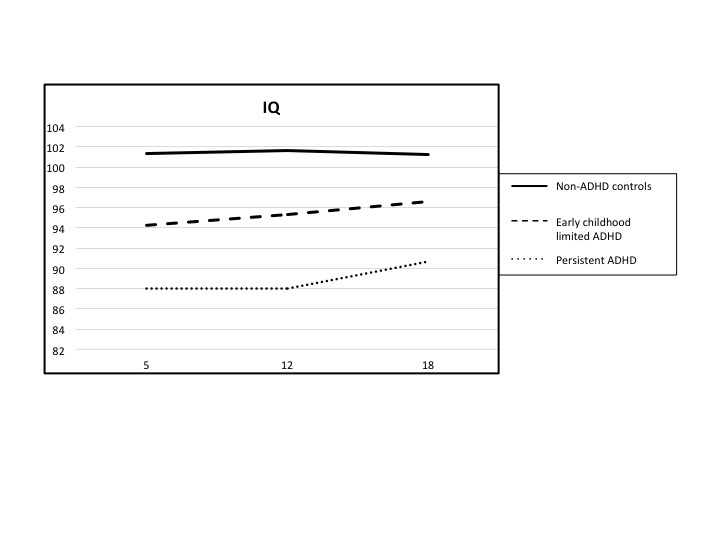

Supplement: supplemental materials [file NIHMS2146799-supplement-supplemental_materials.docx]
